# The association between genetic risk and traditional Chinese medicine syndromes in T2DM patients: A latent class analysis

**DOI:** 10.1097/MD.0000000000042424

**Published:** 2025-08-08

**Authors:** Xiaoyu Wang, Ruiping Pan, Na Zhang, Rong Ma, Huilian Shi, Liqun Wang, Yang Niu

**Affiliations:** a General Hospital of Ningxia Medical University, Ningxia Medical University, Yinchuan, China; b Department of Chinese Medicine, The Second People’s Hospital of Shizuishan, Shizuishan, China; c Department of Chronic and Endemic Disease Control, The Center for Disease Control and Prevention of Guyuan City, Guyuan, China; d Key Laboratory of the Ningxia Ethnomedicine Modernization, Ministry of Education, Ningxia Medical University, Yinchuan, China; e Department of Epidemiology and Statistics, School of Public Health, Ningxia Medical University, Yinchuan, China.

**Keywords:** gene risk, latent class analysis, T2DM patients, traditional Chinese medicine syndromes

## Abstract

Traditional Chinese medicine (TCM) syndromes play a vital role in treating diabetes, and genetics have long been recognized as influcing factor of diabetes. This study aimed to confirm the latent classes of TCM syndromes in type 2 diabetes mellitus (T2DM) patients and to explore the association between genetic risk and those TCM syndromes classes. A total of 1045 T2DM patients from the Ningxia province of China were included in this study. The TCM syndromes scale was used to collect the syndrome differentiation information. Seven gene polymorphisms were assessed using standard procedures. Latent class analysis was used to determine unobserved classes of TCM syndromes. Multinomial logistic regression was employed to examine the relationships between gene risk and classes of TCM syndromes. The optimal number of latent classes was 3, labeled as blood stasis, phlegm dampness and depression heat, and blood deficiency and damp heat type. Almost 12% of the sample belonged to the blood deficiency and damp heat group. Participants with higher levels of gene risk score (RRR = 1.85, 95% CI: 1.18, 2.90) were more likely to have blood deficiency and damp heat type than those with lower levels of gene risk score. The findings provides a molecular biology basis for the objective study of TCM syndromes in T2DM and achieved a combination of macro and micro levels, and also provides a reference for patients to undergo syndrome differentiation and personalized intervention.

## 1. Strengths and limitations of this study

Overall, this study is the first known study to explore the correlation between multi-gene risk scores and traditional Chinese medicine (TCM) syndromes in T2DM patients. We found GRS may increase the risk of the blood deficiency and damp heat type of TCM syndrome rather than the other types. In addition, this study is a multi-center study, which is well representative of T2DM patients in Ningxia.

However, due to some objective factors, this study inevitably has the following limitations. First, a cross-sectional study limits the inference of causal relationships. Second, although a structured and quantitative TCM syndrome scale was employed, avoiding subjectivity in TCM syndrome differentiation is still difficult. Third, although we adjusted for some key covariates during the analysis, there may still be other unmeasured or unknown variables that may affect the results, so more research is needed for further validation.

## 2. Introduction

With the increased life expectancy, unprecedented economic growth and the significant change of lifestyles, China has the largest number of diabetics worldwide, especially type 2 diabetes mellitus (T2DM) patients.^[1]^ The latest epidemiological survey data indicate that the prevalence of T2DM in mainland China is about 12.8%.^[2]^ They are at risk of developing life-threatening complications resulting in reduced quality of life, increased mortality, and higher healthcare costs.^[3]^ While diabetes remains a significant global health challenge, there are many effective prevention strategies and treatment options available. Lifestyle modifications, medication, and regular monitoring can help manage the condition and improve quality of life. Especially, a combination of traditional Chinese medicine (TCM) syndromes and modern medicine may provide new ideas to prevent or treat T2DM by complementing their advantages.

For centuries, TCM has been proven to be highly effective in treating numerous chronic and critical illnesses, including diabetes, which can be traced back to the theories illustrated in the inner canon of yellow emperor.^[4]^ Modern medicine, which focuses on blood glucose regulation, has certain limitations in the treatment of T2DM and its complications. In contrast, TCM, which is characterized by syndrome differentiation or “bian zheng lun zhi” in Chinese^[5]^ and focuses on the concept of holism, has obvious advantages in the treatment of T2DM.^[6]^ “Syndrome” is an abstract generalization of the pathological changes of a disease at a certain phase, which can reveal the essence of the disease in a deeper and more comprehensive way and is the basis of clinical treatment of TCM.^[6]^

In the clinical practice of TCM, the pathogenesis of T2DM involves deficiency syndromes and excess syndromes, and deficiency syndromes are mainly manifested as Qi deficiency, blood deficiency, Yin deficiency, and Yang deficiency, and excess syndromes are primarily embodied as phlegm dampness, heat formation, and damp heat, etc.^[7]^ It is worth noting that usually, If a disease occured or developed，not a single TCM syndrome, but multiple TCM syndromes existed simultaneously, manifesting as complex constitutions. Regarding the complexity of TCM syndromes, a growing number of researches focus on TCM syndromes to provide effective interventions for T2DM. In particular, omics techniques have been used to study TCM syndromes.^[8,9]^

Despite different living environments and habits leading to different TCM syndromes, susceptibility genes in T2DM are also monumental factors affecting the occurrence and development of TCM syndromes. Previous research results show that the TCM syndrome types of diabetes patients are related to the rs290487 polymorphism of the TCF7L2 gene,^[10]^ and the SNP variant T allele of rs7903146 may be related to the newly diagnosed phlegm heat type of diabetes.^[11]^ Studies have found that the ε3/ε4 and ε4/ε4 genotypes and E4 alleles of the Apoε gene are more distributed in patients with Qi deficiency and, blood stasis syndrome and phlegm stasis obstruction syndrome.^[12]^ Some scholars have also conducted small sample studies on the relationship between the red blood cell CR1 molecular gene and the TCM syndromes of diabetes.^[13]^ Moreover, Genetic risk scores (GRS) that leverage additive effects of common risk variants throughout the whole genome can provide risk stratification for complex diseases and with similar effect sizes as traditional risk factors.^[14]^ The recent study reported that GRS could be significant predictors of T2DM development, as well as the diabetes complications development.^[15]^ However, there is still relatively little research on the correlation between TCM syndromes and GRS in T2DM patients. Hence, this study aimed to identify susceptibility genes (rs12505641, rs290487, rs8181588, rs163184, rs2237892, rs5215, rs5219) in T2DM patients and to examine the relationships between GRS and TCM syndromes.

## 3. Materials and methods

### 3.1. Data and participants

**Figure 1.**
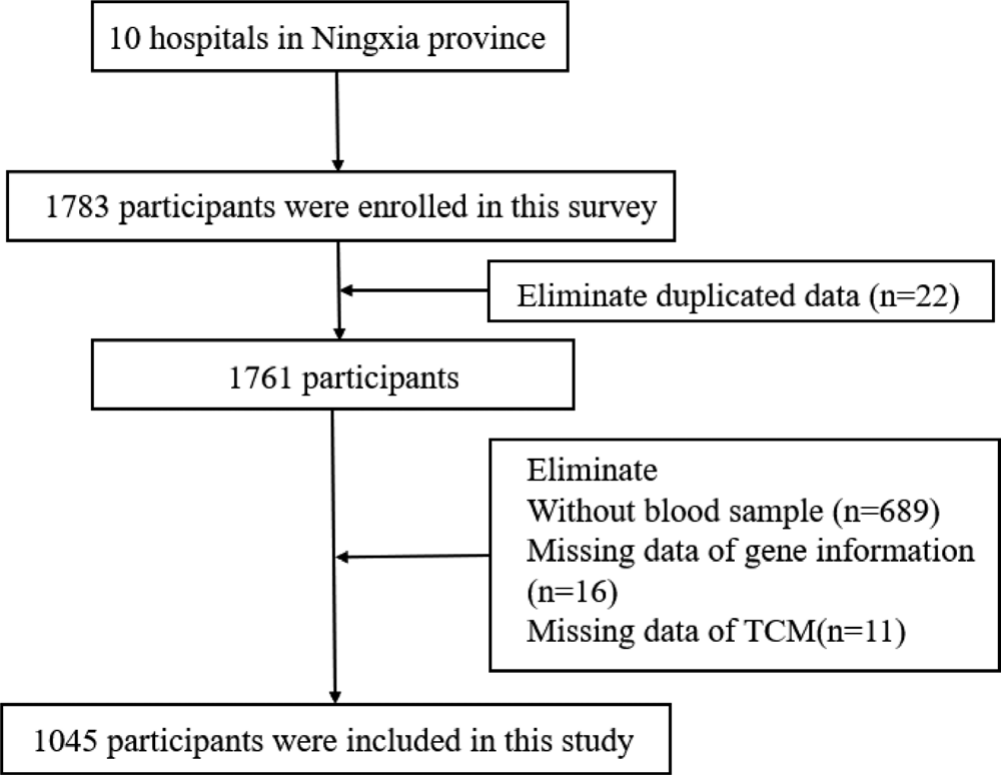
Flowchart for participants selection.

We used the data acquired from 10 hospitals of T2DM patients from August 2019 to November 2020 in Ningxia Province, China. The face-to-face survey was conducted to identify the distribution of TCM syndrome in T2DM patients, and a detailed sampling process can be found elsewhere.^[16]^ The survey used a probability proportionate to size sampling method to collect a representative sample of T2DM patients. The sample comprised 1783 participants in this study; among those, 22 participants were duplicated, 689 were excluded due to not collecting blood samples, 16 missed gene information and 11 were without TCM syndrome information. Finally, the sample of this study included 1045 T2DM patients. A flowchart shows the exclusion of data in Figure 1. This study was approved by the ethical standards of the Institutional Review Board of the Yinchuan Hospital of TCM and with the 1964 Helsinki Declaration and the later amendments or similar ethical standards, and all participants provided informed consent.

### 3.2. Traditional Chinese medicine syndrome

Traditional Chinese medicine syndrome, also known as TCM ZHENG or TCM pattern, is an integral and essential part of TCM theory that helps to guide the design of individualized treatments.^[17]^ In essence, TCM syndrome is a characteristic profile of all clinical manifestations in 1 patient that can be readily identified by TCM doctors.^[18]^ They can, through looking, smelling, questioning, and palpating (or pulse-feeling) to achieve syndrome differentiation. TCM syndrome classification may change as the disease goes through each stage.^[19]^ In this study, we used a TCM syndrome scale to obtain the syndrome information.^[20]^ This scale included 11 TCM syndromes (Yin-deficiency syndrome, Yang-deficiency syndrome, Qi-deficiency syndrome, blood deficiency syndrome, Qi-stasis syndrome, blood stasis syndrome, hyperactivity of liver yang syndrome, phlegm-dampness syndrome, junction-heat syndrome, depression-heat syndrome, and damp-heat syndrome). Each of them is divided into none, light, severe, and heavy, with a score of 0, 2,4, 6, respectively. Cronbach alpha in the present sample is .86, suggesting a reasonably reliable measure for further analysis. In this study, the TCM syndromes were divided into qualitative data with yes or no answers.

### 3.3. Genetic risk score

Using the literature review method and synthesizing literature reports from the past 5 years, the target genes include LOC105374524 rs12505641, TCF7L2 rs290487, KCNQ1 rs8181588, KCNQ1 rs163184, KCNQ1 rs2237892, KCNJ11 rs5215, KCNJ11 rs5219, information of each gene locus can be seen in supplement file (Table S1, Supplemental Digital Content, https://links.lww.com/MD/O889). Single-nucleotide polymorphisms (SNPs) of the rs12505641, rs290487, rs8181588, rs163184, rs2237892, rs5215, rs5219 are detected by a MassARRAY system (Sequenom, San Diego) using the chip-based matrix-assisted laser desorption ionization time-of-flight mass spectrometry technology performed at a genetic testing company in China (BJJH, Beijing). A simple genetic risk score (GRS) were calculated to explore the comprehensive effects of 7 SNPs. GRS calculates the genetic risk score based on the number of alleles carrying mutations. The wild homozygous type is 0 points, the mutant heterozygous type is 1 point, and the mutant homozygous type is 2 points. The sum of all SNP mutations adds up to form the simple genetic risk score, which ranges from 0 to 14 points. The specific calculation formula is as follows: GRS = SNP1 + SNP2 + SNP3 + SNP4 + SNP5 + SNP6 + SNP7. In this study, the subjects were divided into 2 groups based on the median genetic risk score: low genetic risk (<P50) and high genetic risk (≥P50).

### 3.4. Co-variables

Certain covariates formerly shown or hypothesized to be related to TCM syndromes of T2DM patients were evaluated as covariates in our study, including sociodemographic variables, lifestyle behaviors, and disease characteristics. The sociodemographic variables were age, measured in years; gender (male vs female); residence (rural vs urban); marital status (married vs unmarried); education, divided into 4 levels (illiterate, primary school, junior and senior school, and college degree or above); district, divided into 4 groups (Yinchuan, Yinbei, Yinnan, Ningnan). Lifestyle behaviors comprised smoking, alcohol use, and physical activity. Smoking status, alcohol use, and physical activity were divided into “yes/no” groups. Disease characteristics included other chronic diseases, divided into “yes/no” groups; T2DM complication, divided into “yes/no” groups; take medicine, divided into “yes/no” groups; disease duration, measured in years.

### 3.5. Statistical analysis

Mplus version 8 was used for the latent class analyses (LCA).^[21]^ LCA was conducted to investigate the optimal number of latent classes that describe the patients’ TCM syndromes. Models with 1 to 5 profiles were tested. All analyses were conducted using robust maximum likelihood estimation.^[21]^ We used informational criteria Akaike information criterion (AIC), Bayesian information criterion (BIC), and aBIC (BIC using sample size adjustment), in which lower values indicated superior fit. Moreover, we used the Lo–Mendell–Rubin-adjusted likelihood ratio test and the bootstrapped likelihood ratio test to contrast the probability of the number of classes to a solution with fewer classes. Entropy was used to evaluate the classification accuracy, which ranged from 0 to 1, and with higher values indicating greater accuracy, values ≥ 0.8 indicated a good class solution. A *P*-value of < .05 indicates that a particular model fits better than another with 1 fewer classes.^[22]^ The variables included in the study were analyzed in frequency, percentage, mean, and standard deviation. Differences in continuous or categorical variables across TCM syndromes were tested using ANOVA analysis or Chi-square test. Finally, a multinomial logistic regression analysis was performed using SPSS 26.0 (SPSS Inc., Chicago) to confirm factors that predict TCM syndrome in each latent class.

## 4. Results

### 4.1. Latent class analysis

Five latent mixture models were run iteratively, increasing the number of latent classes from 1 to 5. There were 11 TCM syndromes, and the model-fit indices are presented in Table 1. Considering all evaluated fit statistics, tests, and the conceptual separation of latent classes, the model with 3-latent classes was optimal. The LMR was found to be not significantly different in group 6, and entropy is <0.8 in group 5, indicating that a 2 or 3-latent class model was required. Between group 2 and 3, the likelihood is largest for 3 classes. Furthermore, AIC, BIC, and aBIC were the smallest in group 3. While Entropy is higher in group 2, group 3 still provided an ideal value above 0.80. The 3 classes corresponded to the main type of TCM syndromes (“blood stasis”, “phlegm dampness and depression heat”, and “blood deficiency and damp heat”).

**Table 1:** Latent class analysis of TCM syndromes with model-fit results (N = 1045).

	*Npar*	*LL*	AIC	BIC	aBIC	LMRA *P*-value	BLRT *P*-value	Entropy
1-Cluster model	11	−6579.22	13180.44	13234.90	13199.97	–	–	–
2-Cluster model	23	−5695.28	11436.56	11550.45	11477.40	<.001	<.001	0.831
3-Cluster model	35	−5542.98	11155.95	11329.26	11218.10	<.001	<.001	0.815
4-Cluster model	47	−5499.75	11093.50	11326.24	11176.96	.001	<.001	0.754
5-Cluster model	59	−5474.53	11067.06	11359.22	11171.82	.080	<.001	0.715

aBIC = Bayesian information criterion using sample size adjustment, AIC = Akaike information criterion, BIC = Bayesian Information criterion, based on the log likelihood, BLRT = bootstrapped likelihood ratio test, LL = log likelihood, LMRA = Lo–Mendell–Rubin-adjusted likelihood ratio test, Npar = number of parameters in the model, TCM = traditional Chinese medicine.

**Figure 2.**
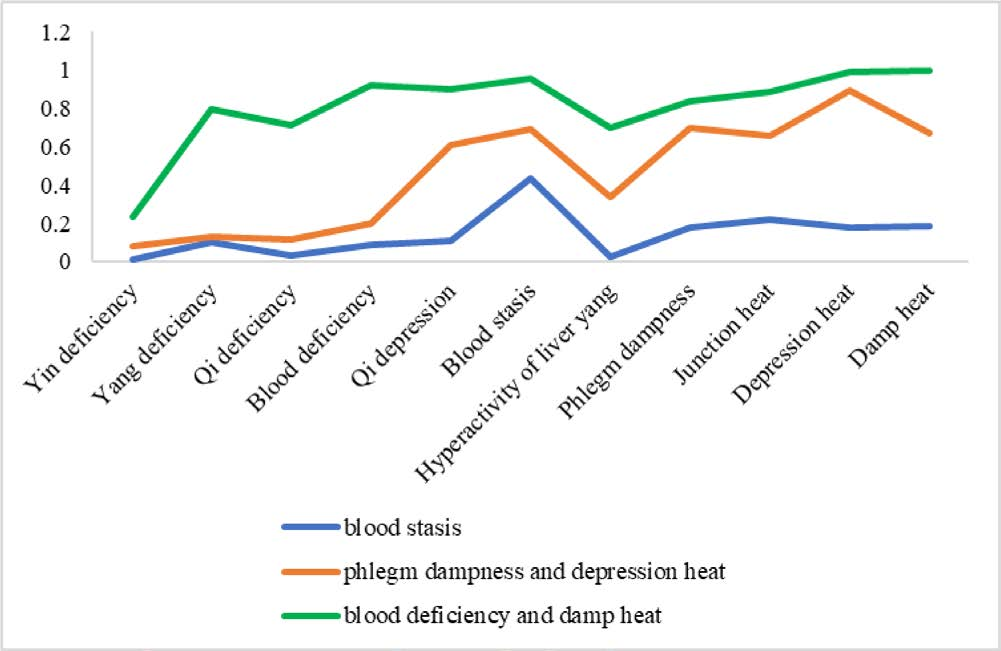
Latent classes of TCM syndromes. TCM = traditional Chinese medicine.

Figure 2 exhibits the cluster-specific estimated probabilities of TCM syndromes for the 3-cluster model. In class1, conditional probability for TCM syndromes was 0.011 for Yin deficiency, 0.101 for Yang deficiency, 0.035 for Qi deficiency, 0.091 for blood deficiency, 0.110 for Qi depression, 0.436 for blood stasis, 0.025 for hyperactivity of liver yang, 0.181 for phlegm dampness, 0.223 for junction heat, 0.175 for depression heat and 0.183 for damp heat. The class had a higher level of blood stasis syndromes and was named the “blood stasis” type. In class 2, conditional probability for TCM syndromes was 0.078 for Yin deficiency, 0.130 for Yang deficiency, 0.113 for Qi deficiency, 0.201 for blood deficiency, 0.612 for Qi depression, 0.690 for blood stasis, 0.339 for hyperactivity of liver yang, 0.702 for phlegm dampness, 0.660 for junction heat, 0.893 for depression heat and 0.673 for damp heat. The class had a higher level of phlegm dampness and depression heat syndromes and was named the ‘phlegm dampness and depression heat’ type. In class 3, conditional probability for TCM syndromes was 0.233 for Yin deficiency, 0.795 for Yang deficiency, 0.710 for Qi deficiency, 0.924 for blood deficiency, 0.899 for Qi depression, 0.958 for blood stasis, 0.700 for hyperactivity of liver yang, 0.840 for phlegm dampness, 0.885 for junction heat, 0.990 for depression heat and 1.000 for damp heat. The class had a higher level of blood deficiency and damp heat syndromes and was named the “blood deficiency and damp heat” type.

### 4.2. Distribution of SNP loci and Hardy Weinberg equilibrium test

The distribution of SNP genotypes and analysis of Hardy Weinberg equilibrium was displayed in Table 2. The genotype frequencies of 7 SNP loci in the control group all met the Hardy Weinberg genetic balance test, and the results indicated that the included population was representative.

**Table 2:** Distribution of SNP genotypes and analysis of Hardy Weinberg equilibrium.

SNP	Genotypes (N)	Reference (class 1) group		χ^2^	*P*_HWE_
		Actual frequency	Theoretical frequency		
	AA (112)	61	59.5		
rs12505641	AG (439)	229	233.2	0.06	0.970
	GG (494)	265	262.4		
	CC (160)	92	85		
rs290487	CT (498)	256	264.5	0.43	0.805
	TT (387)	207	205.5		
	TT (457)	255	242.7		
rs8181588	CT (459)	233	243.8	0.57	0.752
	CC (129)	67	68.5		
	TT (276)	150	146.6		
rs163184	GT (506)	266	268.7	0.05	0.975
	GG (163)	139	139.7		
	TT (71)	38	37.7		
rs2237892	CT (426)	213	226.2	0.67	0.716
	CC (548)	304	291		
	TT (366)	201	194.4		
rs5215	CT (487)	261	258.6	0.55	0.761
	CC (192)	93	102		
	TT (197)	98	104.6		
rs5219	CT (518)	279	275.1	0.30	0.863
	CC (330)	178	175.3		

Class 1: blood stasis type.

SNP = single-nucleotide polymorphisms.

### 4.3. Characteristics of 3 clusters

Table 3 shows the characteristics of 3 classes. The average age was 57.9 (SD = 12.0) years. Slightly more than half (55.5%) were male, over a quarter (16.5%) were illiterate, and about 93.6% were married. The prevalence of high GRS was 55.3%. living in rural areas, with lower education levels, living in remote district, short disease duration, physical inactivity, and with high levels of GRS were distributed more in class 3.

**Table 3:** Characteristics and TCM syndrome distribution of the clusters.

Variables	Total (n = 1045)	Class 1 (n = 555)	Class 2 (n = 368)	Class 3 (n = 122)	*P*-value
Age, mean (SD), yr	57.9 (12.0)	57.2 (11.7)	59.3 (12.1)	57.0 (12.6)	.027
Gender, male, n (%)	580 (55.5)	294 (53.0)	221 (60.1)	65 (53.3)	.092
Residence, urban, n (%)	719 (68.8)	417 (75.1)	252 (68.5)	50 (41.0)	<.001
Marital status, married, n (%)	978 (93.6)	515 (92.8)	348 (94.6)	115 (94.3)	.532
Education level, n (%)					
Illiterate	172 (16.5)	82 (14.8)	55 (14.9)	35 (28.7)	.001
Primary	202 (19.3)	102 (18.4)	70 (19.0)	30 (24.6)	
Junior and Senior	416 (39.8)	231 (41.6)	147 (39.9)	38 (31.1)	
College degree or above	255 (24.4)	140 (25.2)	96 (26.1)	19 (15.6)	
District, n (%)					
Yinchuan	497 (47.6)	319 (57.5)	163 (44.3)	15 (12.3)	<.001
Yinbei	146 (14.0)	68 (12.3)	69 (18.8)	9 (7.4)	
Yinnan	352 (33.7)	131 (23.6)	125 (34.0)	96 (78.7)	
Ningnan	50 (4.8)	37 (6.7)	11 (3.0)	2 (1.6)	
Smoking, yes, n (%)	255 (24.4)	151 (27.2)	223 (26.5)	27 (22.1)	.077
Alcohol use, yes, n (%)	261 (25.0)	146 (26.3)	649 (22.7)	36 (29.5)	.118
Physical activity, yes, n (%)	696 (66.6)	395 (71.2)	579 (68.9)	67 (54.9)	.001
Other chronic diseases, yes, n (%)	684 (65.5)	365 (65.8)	238 (64.7)	81 (66.4)	.918
T2DM complications, yes, n (%)	618 (59.1)	337 (60.7)	224 (60.9)	57 (46.7)	.012
Take medicine, yes, n (%)	873 (83.5)	469 (84.5)	302 (82.1)	102 (83.6)	.619
Disease duration, mean (SD), yrs	8.6 (7.6)	9.3 (7.7)	8.3 (7.6)	6.5 (6.3)	<.001
GRS, high level, n (%)	578 (55.3)	297 (53.5)	201 (54.6)	80 (65.6)	.047

*P*-value is presented from ANOVA test or Chi-square test. Class 1 = blood stasis type, class 2 = phlegm dampness and depression heat type, class 3 = blood deficiency and damp heat type.

GRS = genetic risk score, SD = standard deviation, T2DM = type 2 diabetes mellitus, TCM = traditional Chinese medicine.

### 4.4. Multinomial logistic regression analysis

As shown in Table 4, after controlling the confounders, compared with blood stasis type, T2DM patients with a higher level of GRS were more likely to have a blood deficiency and damp heat type of TCM syndromes than those with a lower level of GRS. The relative risk ratio for blood deficiency and damp heat type was 1.85 (95% CI: 1.18–2.90). However, this relationship did not exist between phlegm dampness and depression heat and blood stasis type.

**Table 4:** Relative risk ratios for the relationships between GRS and clusters from multinomial logistic regression analyses (n = 1045).

	Class 2 (vs class 1)		*P*-value	Class 3 (vs class 1)		*P*-value
	RRR	(95% CI)		RRR	(95% CI)	
Age	1.03	(1.01,1.04)	<.001	1.01	(0.98,1.03)	.320
Gender	1.90	(1.34,2.66)	<.001	0.95	(0.56,1.67)	.852
Marital status	1.28	(0.72,2.27)	.452	1.12	(0.44,2.88)	.810
Education (illiterate)	0.58	(0.33,1.01)	.091	0.91	(0.68,1.21)	.526
Residence	0.73	(0.53,1.07)	.753	0.45	(0.26,0.76)	.003
District (Yinnan)	1.15	(0.98,1.34)	.084	1.32	(2.80,2.99)	<.001
Smoking	0.63	(0.43,0.92)	.016	0.66	(0.35,1.25)	.204
Alcohol use	0.80	(0.55,1.16)	.234	2.84	(1.53,5.25)	.001
Physical activity	0.71	(0.53,0.95)	.020	0.45	(0.28,0.68)	<.001
Disease duration	0.97	(0.95,0.99)	.004	0.95	(0.92,0.99)	.014
T2DM complications	1.11	(0.82,1.49)	.510	0.70	(0.44,1.11)	.128
Other chronic diseases	0.88	(0.65,1.19)	.423	1.06	(0.65,1.73)	.778
Take medicine	0.88	(0.61,1.28)	.524	1.28	(0.69,2.39)	.429
GRS	1.05	(0.80,1.37)	.729	1.85	(1.18,2.90)	.007

Class 1 = blood stasis type, class 2 = phlegm dampness and depression heat type, class 3 = blood deficiency and damp heat type.

95% CI = 95% confidence interval, GRS = genetic risk score, RRR = relative risk ratios.

## 5. Discussion

The purpose of this study was to investigate latent groups of 11 TCM syndromes and to identify associations with group membership by GRS. The analysis provided reasonable empirical evidence to support the hypothesis that latent groups of TCM syndromes exist. To our knowledge, this is the first study to use LCA to distinguish unobserved groups of TCM syndromes and demonstrate evidence of associated GRS.

This study found 3-latent classes of TCM syndromes in T2DM patients, namely, blood stasis type (class 1), phlegm dampness and depression heat type (class 2), and blood deficiency and damp heat type (class 3), represented indicators of TCM syndromes. Of the 3 classes, the first class was the largest (52.5%), representing those who reported the main TCM syndromes was blood stasis type. This class was characterized by high probability of blood stasis syndrome. Because this type has the highest proportion among the 3 classes, so blood stasis type can represent the general characteristics of individuals and can be used as the reference standard in research on TCM syndromes in T2DM patients. Class 2 and class 3 accounted for 35.6% and 11.9%, respectively. This manifested that 88.1% of TCM syndromes of T2DM patients belonged to the blood stasis or phlegm dampness and depression heat type, even if various kinds of TCM syndromes coexisted. In the bargain, we found that in class 1, blood stasis syndrome was higher in this class than in the other 2 classes. Inversely, Yin deficiency and hyperactivity of liver yang syndrome were lower in this type group. In the class 2, phlegm dampness and depression heat were the main manifestations of the syndrome. In the same way, in class 3, blood deficiency and damp heat syndrome were higher in this class than in the other 2 classes. The emerging evidence showed that Yin deficiency, Qi deficiency, and Phlegm dampness or damp heat syndromes were more frequent than other syndromes among T2DM patients.^[23]^ Our study also found that participants with physical activity were less likely to have phlegm dampness and depression heat, and blood deficiency and damp heat type of TCM syndromes. The possible reason may be that physical activity can promote blood circulation, keeping people in a healthy state and a happy mood,^[24]^ and further to reduce the possibility of depression and obesity. Furthermore, this study found that those with alcohol use behavior were more likely to have a blood deficiency and damp heat type. This result was consistent with a previous study that reported that alcohol use could lead to lung yin depletion, dampness and heat trapping the spleen, endogenous phlegm turbidity, poor Qi circulation, and delayed blood circulation.^[10]^

Another aim of this study was to explore the relationship between GRS and the class of TCM syndrome. The results showed that participants with higher levels of GRS were more likely to emerge in blood deficiency and damp heat type. Although biological characteristics such as genetic variation cannot be altered, for high-risk populations, taking early targeted preventive measures may result in greater health benefits. There is currently limited research exploring the association between multi-gene GRS and TCM syndromes. The previous study has suggested that the T2DM patients carrying TC genotype at rs290487 locus is mainly characterized by Qi stasis and blood deficiency, as well as spleen deficiency and phlegm dampness syndrome.^[24]^ This study explores the genetic risk score of TCM syndrome in T2DM patients, which may provide new clues for the objective study of TCM syndromes. However, the possible mechanism between GRS and TCM syndromes of T2DM patients still needs further clarification.

## 6. Conclusions

This is the first study to investigate the relationship between GRS and TCM syndromes using LCA in a sample of T2DM patients. The results suggested that GRS may increase the risk of a blood deficiency and damp heat type of TCM syndrome. This provides a molecular biology basis for the objective study of TCM syndromes in T2DM and also provides a reference for patients to undergo syndrome differentiation and personalized intervention.

## Acknowledgments

We thank all hospitals and participants for their cooperation.

## Author contributions

**Data curation:** Xiaoyu Wang, Ruiping Pan, Na Zhang, Rong Ma, Huilian Shi, Liqun Wang, Yang Niu.

**Formal analysis:** Xiaoyu Wang, Ruiping Pan, Na Zhang, Rong Ma, Huilian Shi, Liqun Wang, Yang Niu.

**Methodology:** Yang Niu.

**Software:** Yang Niu.

**Writing – original draft:** Xiaoyu Wang, Yang Niu.

**Writing – review & editing:** Xiaoyu Wang, Yang Niu.

## Supplementary Material


