# Supplementary material for: The association between genetic risk and traditional Chinese medicine syndromes in T2DM patients: A latent class analysis
Source: Medicine (Baltimore). 2025 Aug 8;104(32):e42424. doi: 10.1097/MD.0000000000042424 (PMC12338265; doi:10.1097/MD.0000000000042424)
Supplement: Supplementary file 1 [file medi-104-e42424-s001.pdf]

Table S information of SNP in LOC105374524, TCF7L2, KCNQ1 and KCNJ11 gene

SNP	Gene	Location	Mutation	Functional domain
rs12505641	LOC105374524	4:23487269	G:A	intron
rs290487	TCF7L2	10:113149972	C:T	intron
rs8181588	KCNQ1	11:2810311	T:C	intron
rs163184	KCNQ1	11:2825839	T:G	intron
rs2237892	KCNQ1	11:2818521	C:T	intron
rs5215	KCNJ11	11:17387083	C:T	Missense
rs5219	KCNJ11	11:17388025	C:T	5'-UTR/ Missense/ intron
